# Considering Long-Acting Synthetic Cannabidiol for Chronic Pain: A Narrative Review

**DOI:** 10.7759/cureus.81577

**Published:** 2025-04-01

**Authors:** Paul J Christo, Eugene Vortsman, Christopher Gharibo, Jo Ann K LeQuang, Joseph V Pergolizzi

**Affiliations:** 1 Division of Pain Medicine, Department of Anesthesiology and Critical Care Medicine, Johns Hopkins University School of Medicine, Baltimore, USA; 2 Department of Emergency Medicine, Northwell Health, Long Island Jewish Medical Center, New York, USA; 3 Department of Pain Management, NYU Langone Health, New York, USA; 4 Scientific Communications, NEMA Research, Inc., Naples, USA; 5 Department of Pain Medicine, NEMA Research, Inc., Naples, USA

**Keywords:** cannabidiol (cbd), cannabinoid-based therapies, chronic pain management, multimodal pain control, synthetic cannabidiol

## Abstract

Chronic pain is prevalent and challenging to treat. Cannabinoids, in particular cannabidiol (CBD), have been evaluated as analgesics without the issues of tolerance or dependence. Side effects tend to be mild and infrequent. These products have multiple routes of administration and composition, and some are available over the counter, allowing pain patients to self-medicate. Most self-medicated CBD are plant-derived extracts administered as either oils, pills, or by inhalation. During the early 1960s, CBD was chemically synthesized for the first time, but it was not yet approved for medical use; synthetic CBD has been and continues to be studied in clinical trials for numerous indications, including chronic pain, neuropathic pain, and pain in cancer. However, studies are often small, populations heterogeneous, and some results are equivocal. Research is lively, with over 60 studies reported on ClinicalTrials.gov. Multimodal CBD therapy may hold promise, particularly in combination with palmitoylethanolamide. Greater patient education and training for physicians and other healthcare providers are needed along with more comprehensive studies. Considering the problem of chronic pain, further intensive study of synthetic CBD for pain control is warranted to meet this unmet clinical need. This is particularly important in the context of long-lasting administration methods that enable easy dosing and support long-term use for patients dealing with persistent and often debilitating symptoms.

## Introduction and background

Analgesic mainstays of nonsteroidal anti-inflammatory drugs (NSAIDs) and opioids are limited in their ability to manage chronic pain in the millions of people around the world with chronic pain or life-altering “high-impact chronic pain” that confers disability [1,2]. The conundrum of chronic pain relief is best conveyed by the fact that 22.1% of the adult US population has received a prescription for an opioid pain reliever in the past three months [3], despite the fact the role of opioids in long-term pain control is controversial [4] and side effects, dependence, tolerance, and the risk of use disorder are well described in the literature and by public health agencies [5,6].

The earliest reports of medical marijuana date back to 2700 B.C. in China, although the plant likely did not arrive in the West until the first century [7]. Cannabis products were legal and widely used in early America and did not become illegal until 1937; it was the American Medical Association that argued before Congress to keep marijuana legal because of its nearly irreplaceable medical value [8]. The *Cannabis sativa* plant has been employed to treat a broad range of medical conditions: chronic pain, peripheral neuropathy, nausea and emesis, gastrointestinal disorders, bone loss, hypertension, seizures, and hyperglycemia. It has been suggested that cannabidiol (CBD), one of the constituents of the plant may be able to inhibit the growth of cancer cells [9]. Its antispastic properties show important potential in treating multiple sclerosis; it has been advocated to reduce intraocular pressure characteristic of glaucoma [10].

The role of CBD and its sister constituent tetrahydrocannabinol (THC) is the best studied of the more than 500 constituents of the *C. sativa* plant [9]. Preclinical studies of CBD and THC, alone or in combination, have shown great promise in treating chronic and neuropathic pain, but clinical studies are fewer and have not yet produced the same body of robust evidence [11].

CBD and THC were first synthesized in 1965 in the laboratory of Professor Raphael Mechoulam [12]. Since then, licit synthetic cannabinoids, including the THC analogs dronabinol (marketed as Marinol) and nabilone (marketed as Cesamet), have been approved in the United States for use as antiemetic treatment with off-label application in pain management. In contrast, synthetic forms of CBD have not yet received approval for medical use, but are available as a synthetic active pharmaceutical ingredient for research purposes. Illicit synthetic THC forms are available on the street for their strong psychoactive effects. Despite the fluctuating legal status of THC and CBD across the United States, approximately 14% of Americans report using CBD for therapeutic purposes, with an estimated 40% utilizing it specifically for pain management in 2019. Similar to plant-derived THC, licit synthetic cannabinoids target the endocannabinoid system by binding to the CB-1 cannabinoid receptor [13-17].

The objective of this narrative review is to explore the potential role of CBD, THC, and their synthetic products for the long-term treatment of chronic pain in humans.

Methods

On November 18, 2023, PubMed was searched for “CBD, chronic pain” (387 results), “cannabinoid, chronic pain” (1,604 results), and “synthetic cannabinoid (4,472 results). No delimiters were applied. For a table presentation of studies, a search was conducted on the same day for “cannabinoid, chronic pain” and “synthetic cannabinoid, chronic pain” but limited to “clinical trial” or “randomized clinical trial” which yielded 61 and 101 results, respectively. When those results were further filtered for “associated data,” there were 29 results for “synthetic cannabinoids, chronic pain” and 23 for “cannabinoids, chronic pain.” The references of important papers in this search were checked and Google Scholar was also used.

## Review

The endocannabinoid system, a complex lipid signaling system in the central and peripheral nervous systems, promotes homeostasis in the body; the disruption and/or dysfunction of the endocannabinoid system may have been associated with certain chronic inflammatory conditions, immune system disorders, gut dysbiosis, migraines and cluster headaches, and certain mood disorders [18-21]. The mechanism of action of CBD is at some levels distinct from that of endogenous cannabinoids, as it can activate the serotoninergic system via the 5-HT-1A receptor, coupled to the Gi protein [22].

There are two known cannabinoid receptors (CB), numbered 1 and 2. CBD is a negative allosteric modulator at the CB-1 receptor and a partial agonist at the CB-2 [23]. Recent studies indicate that CBD may also act as a negative allosteric modulator of the CB-2 receptor, given its weak affinity for the receptor’s orthosteric site [24]. Allosteric modulators do not exert intrinsic efficacy, but enhance or reduce the receptor’s response to orthosteric ligands, which bind to the same site as the endogenous ligand. This allows a CB receptor to send signals without the risk of developing tolerance, dependence, or desensitization [25]. Allosteric ligands bind to the topographical portions of the receptor at different places than the binding sites, permitting them to modulate orthosteric ligands [26] Allosteric modulators may be described as positive (increasing signal activity), negative (decreasing signal activity), or silent (no change to signal activity) [25]. Thus, the endocannabinoid system consists of CB-1 and CB-2 receptors and lipid mediators known collectively as endocannabinoids. The two main endocannabinoids are anandamide and 2-arachidonoylglycerol\, along with related enzymes to synthesize and degrade them and their transport systems [25,27]. While THC primarily exerts its effect through orthosteric binding to CB-1 receptors, CBD, which has a weak affinity to the orthosteric site of the CB-2 receptor, is suggested to mediate its biological activities via alternative mechanisms. These include interactions with the transient receptor potential vanilloid 1, alpha-1 and alpha-1-beta glycine receptors, G protein-coupled receptor 55 (GPR55), serotonin 5-HT-1A and peroxisome proliferator-activated receptor gamma (PPAR-γ) [17]. Besides a direct interaction of CBD with specific receptors, it enhances the activity of endocannabinoids through modulating their degrative mechanism [28]. While two main cannabinoid receptors are recognized, it is possible that GPR55, an orphan G-protein coupled receptor, may also act as a kind of cannabinoid receptor as well. Serotonin (5HT-3) and N-methyl-D-aspartate may further play a role in cannabinoid signaling. Endocannabinoids have been observed to interact promiscuously with receptors and dynamic interactions between substrates and metabolites have been observed [18,19]. The term “endocannabinoidome” has been proposed to better understand the connections the endocannabinoid system has with other governing systems in the body [18]. See Table 1.

**Table 1:** Role of the cannabinoid system in pain management While two main cannabinoid receptors are recognized, it is possible that GPR55, an orphan G-protein coupled receptor, may also act as a kind of cannabinoid receptor as well. Serotonin (5HT-3) and N-methyl-D-aspartate receptors may further play a role in cannabinoid signaling pathways. 2-AG, 2-arachidonoylglycerol; AEA, anandamide; CB-1, cannabinoid receptor type 1; CB-2, cannabinoid receptor type 2; CBD, cannabidiol; GABA, gamma-aminobutyric acid; GPR55, G protein-coupled receptor 55; PKA, protein kinase A; PNS, peripheral nervous system; PPAR-γ, peroxisome proliferator-activated receptor gamma; ROCK, Rho-associated, coiled-coil containing protein kinase; THC, tetrahydrocannabinol; TRPV1, transient receptor potential vanilloid 1 [9,25,29-38]

	CB-1 receptors	CB-2 receptors	Other receptors (TRPV1, GPR55, 5HT1A, and PPAR-γ)	Effect on pain
Found mainly in these cells	Presynaptic neurons	Immune cells		CB-2 receptors may influence pain processing, while CB-1 receptors primarily manage the psychological and emotional aspects of pain.
	Neurons in the dorsal root ganglion	Nonneuronal cells (microglia, astrocytes, and macrophages)		
	Smooth muscle cells	Peripheral tissue		
	Adipocytes	Endothelial cells		
	CNS more than PNS	PNS more than CNS		
Activation	Inhibits adenylyl cyclases	Inhibits adenylyl cyclases	Ligand-gated ion channels, leading to the generation of an action potential that signals pain	CB-1 receptors modulate calcium levels by utilizing mitochondria as reservoirs. TRPV1 plays a central role in pain sensation, particularly in nociception. GPR55 is significantly involved in inflammatory pain, potentially modulating pain signaling at both peripheral and central levels. PPAR-γ, a nuclear receptor, regulates various genes related to metabolism and immune response. Meanwhile, the 5HT1A receptor, part of the serotonergic system, influences mood, behavior, and sensory perception, including pain.
	Inhibits voltage-activated Ca2+ channels	No effect on ion channels	Modulates key sensory signaling components such as Ca+2, RhoA/ROCK, and PKA/cAMP	
	Inhibits GABA and glutamate		Inhibition of proinflammatory cytokines	
Effect on inflammatory pain	Does not reduce inflammatory pain	Reduces inflammatory pain	Reduces inflammatory and noninflammatory pain	
Affinity	THC, CBD (allosteric), AEA, and 2-AG	CBD (allosteric or weak orthosteric), 2-AG, and AEA	CBD, AEA, and 2-AG	

The endocannabinoid system plays a role in pain modulation and works at the supra-spinal, spinal, and peripheral levels in ways that have not yet been entirely elucidated [36]. CBD and THC interact with the endocannabinoid system in different ways (both direct and indirect interactions). CBD has less psychoactive effect than THC and combination products of CBD and THC produce less psychoactive effect than THC alone because the negative allosteric modulation causes CBD to compete with THC and inhibit the latter’s ability to bind to receptors [39].

The extent of the first-pass metabolism of CBD in the liver influences its pharmacokinetic (PK) profile and, in particular, its bioavailability. Thus, orally administered CBD is extensively metabolized before it enters the systemic circulation, accounting for its estimated bioavailability of approximately 6-20% [40,41]. CBD is metabolized via the cytochrome-P (CYP)-3AF substrate and inhibits the CYP2C19 enzyme [39]. It is sequestered in adipose tissue and highly vascularized tissue because it is a small lipophilic molecule [41]. For this reason, the absorption, distribution, metabolism, and elimination of CBD are impacted by dose, dosing schedule, and diet [42].

CBD may exert an anti-inflammatory effect as well, which is not evident in THC alone [22]. Inflammatory pain occurs due to an inflammatory immune response which may initiate as a protective mechanism but become maladaptive and a source of ongoing, even chronic pain. The prolonged activation of sensory neurons can lead to neuropathic pain [22].

Chronic noncancer pain

While patients may perceive chronic and acute pain as the same thing, they are mechanistically distinct, with chronic pain involving centralization and aberrant signal processing unrelated to an actual injury. In this way, chronic pain is maladaptive, while acute pain maps the trajectory of tissue healing following an injury or disease. In fact, current thinking considers chronic pain as a disease unto itself, apart from any underlying injury or condition [43]. Unlike acute pain, chronic pain should be viewed in a biopsychosocial context, meaning that pain treatment must consider holistic factors [44-46]. Pharmacologically, the treatment of chronic pain is challenging because it demands long-term exposure of the patient to an analgesic regimen, which limits the use of NSAIDs and opioids for such conditions [47-50]. These limitations have given rise to interest in CBD for treating chronic noncancer pain. In Australia, about 65% of prescriptions for medicinal forms of cannabis are for the treatment of chronic noncancer pain [51].

Chronic pain is often treated with a combination of direct disease treatment, and nonpharmacologic, pharmacologic, and interventional treatments that may ultimately include stronger agents, including opioids [52]. Although the now iconic World Health Organization (WHO), “pain ladder” was originally proposed for managing cancer pain, the idea of treating pain incrementally based on pain intensity has carried over to chronic pain. Of course, the “incremental analgesic” approach of the WHO pain ladder has been critiqued, because it considers only pain intensity rather than other factors, such as inflammation or disability, and ignores the more global and psychological experience of pain [53]. Adjuvant medications, notably antidepressants and gabapentinoids can supplement an analgesic regimen for chronic pain and may be needed to address a neuropathic component, typical in chronic pain [54]. It should be noted while people with chronic pain often suffer from comorbid depression, antidepressants at specific low doses confer an analgesic benefit, distinct from their anti-depressive characteristics [55].

Finally, there are other treatments for chronic pain beyond pharmacological measures including physical therapy, occupational therapy, exercise, and lifestyle modifications, such as sleep hygiene and weight loss. Interventional pain medicine may employ open or minimally invasive surgery, nerve blocks, injections, neuromodulation devices, intrathecal targeted drug delivery, or other means to address pain [56-58]. Complementary and alternative medicine (CAM) has also been used to address pain and includes massage, chiropractic, and acupuncture [59-62]. It is often used as an adjunctive treatment along with a more conventional pharmacologic approach. Not all patients consider CAM or nonpharmacological approaches, and some who may benefit from exercise or occupational therapy become nonadherent [63].

There is no “gold standard” for managing chronic pain and the multidisciplinary approaches sometimes recommended in guidelines are not always available in real-world clinical practice [64]. The pharmacological approaches have known drawbacks, such as side effects, and potential toxicity at any dose, and there is the risk of tolerance, use disorder, and immune system disruptions with opioids [65-68]. In an eight-week study of 97 pain patients taking opioid analgesics for at least one year, 53% were able to reduce or eliminate their use of opioids following the introduction of CBD-rich hemp extract, suggesting that CBD could reduce opioid consumption without sacrificing analgesic benefit [69]. A survey of patients at a pain management clinic found that 71% thought CBD was helpful for managing pain and 54% reported their use of CBD helped them reduce the consumption of opioid analgesics [70].

To date, there have been few clinical studies about the potential analgesic action of CBD in chronic noncancer pain, and of those published, most were small studies with design limitations [13]. Furthermore, differences in pain mechanisms in various conditions make it challenging to find robust evidence relating broadly to chronic noncancer pain and CBD. A systematic review of medical cannabinoids for all indications evaluated 152 randomized clinical trials with a total of 12,123 participants and reported that cannabinoids were effective for several medical indications, including chronic pain, and merit further evaluation [71]. While no CBD-only products have been approved to market for pain control, evidence suggests that the combination product of CBD and THC offers analgesic benefits [72]. Currently, available cannabinoid-based analgesics include synthetic THC alone, and plant-derived purified CBD:THC in combination. These products are used in a variety of formulations, including the oromucosal spray combining CBD and THC (nabiximol, trade name Sativex^®^, GW Pharmaceuticals Limited, Cambridge, United Kingdom). These products are being studied with respect to their analgesic benefits but have sometimes also improved the quality of sleep, reduced muscle spasms, and spasticity in patients with multiple sclerosis, and improved bladder control in multiple sclerosis [73]. See Table 2.

**Table 2:** Studies of synthetic CBD in various populations The studies were grouped by type of pain syndrome and arranged in alphabetic order by surname of the first author. Studies dealt with chronic pain but were not necessarily long-term studies. AE, adverse event; CBD, cannabidiol; MS, multiple sclerosis; NRS, Numerical Rating Scale; RT, randomized trial; THC, tetrahydrocannabinol [69,74-83]

Study	Indication	Patients	Design	Duration	Results
Cancer pain					
Good et al. (2020) [74]	Cancer pain at end of life	21	Impact of CBD and THC on symptom burden	Four weeks	43% responded, doses well tolerated
Johnson et al. (2010) [75]	Intractable cancer pain	177	Comparison of THC:CBD spray vs. THC alone vs. placebo	Two weeks	THC:CBD was significantly superior in reducing pain compared to placebo, while THC alone was similar to placebo.
Portenoy et al. (2012) [76]	Chronic, poorly controlled cancer pain	263	THC:CBD vs placebo for pain	Five weeks	No significant difference between groups for pain based on a 30% responder rate, but the intervention was significantly superior to the placebo for average daily pain scores over the course of five weeks.
Chronic pain					
Capano et al. (2020) [69]	Chronic pain	131	Impact on opioid consumption	Eight weeks	53% reduced or eliminated their use of opioids at eight weeks.
Ueberall et al. (2019) [77]	Severe chronic pain	30,228 retrospective cases	THC:CBD spray as an add-on	12 weeks of open-label data	15% improved on all nine symptom factors and 56% had ≥50% improvement in 5/9 factors, especially control of neuropathic pain.
van de Donk et al. (2019) [78]	Chronic pain with fibromyalgia	25 women	Inhaled cannabis for pain; four-way crossover study	Response measured for 3 hours per dose	No treatment (single inhalation) was superior to placebo.
Neuropathic or neurogenic pain					
Rog et al. (2005) [79]	Central neuropathic pain in MS	66	THC:CBD over two years	Five-week RT followed by an open-label period. Mean open-label treatment was 463 days; 44% of open-label patients were treated with a median of 845 days	Mean NRS was 2.9 (range: 0-8.0) at two years, and 92% had ≥1 treatment-related AE (most mild to moderate). No evidence of tolerance at two years.
Serpell et al. (2014) [80]	Peripheral neuropathic pain	303	THC:CBD spray vs placebo	15 weeks	Intervention was superior to placebo at a 30% responder rate based on NRS
Wade et al. (2003) [81]	Intractable neurogenic symptoms	24 pt with MS, spinal cord injury, brachial plexus damage, or limb amputation	Plant-derived cannabis extracts vs placebo in a series of single-patient crossover studies	Two-week treatment period	Analgesia was superior to placebo and some patients had other symptomatic improvements (bladder control, muscle spasms, and spasticity)
Transplantation					
Cuñetti et al. (2018) [82]	Pain following kidney transplantation	Seven patients who requested CBD	CBD	Three weeks	Two patients reported total improvement in pain, four partial improvement, and one had no change over 15 days
Acute pain					
Chrepa et al. (2024) [83]	Dental pain	61 patients with emergency acute dental pain	Oral CBD: patient randomized to CBD 10 mg/kg, CBD 20 mg/kg, placebo	Three hours	Both CBD grounds had significant reductions in acute pain

Systematic reviews

Several systematic reviews and meta-analyses explored CBD analgesics for use in chronic pain. Fitzcharles et al. evaluated three studies (n = 159) for the analgesic benefit of CBD products in patients suffering pain associated with rheumatic diseases. Findings were equivocal, in part because studies were at risk for bias. However, CBD was well tolerated in these patients [84]. In a systematic review and meta-analysis encompassing 96 randomized clinical trials with a total of 26,169 participants suffering from nonmalignant forms of chronic pain (median age: 58 years), opioids were shown to be effective analgesics [85]. However, the drawbacks of opioid analgesics are well known and include tolerance, potentially treatment-limiting opioid-related side effects, and the risk of opioid use disorder [6].

In a systematic review and meta-analysis of 25 clinical studies of chronic pain conditions with a total of 14,835 participants, cannabinoids offered analgesic benefits but the analysis explored short-term use only in chronic pain and could not provide data related to prolonged exposure to CBD products [86]. A meta-analysis of 16 studies with 1,750 subjects with chronic neuropathic pain likewise could not offer long-term findings but found that cannabis-based medical products resulted in both analgesic benefits and certain adverse events [87].

In a systematic literature review of 59 studies that used a variety of cannabinoid products to treat gynecologic pain, 61-95.5% reported pain relief. Conditions reported in this analysis included chronic pelvic pain, endometriosis, bladder pain, and gynecologic cancer pain [88]. A systematic review of medical cannabinoids (CBD, dronabinol, nabilone, and nabiximol) found evidence in support of their use for a multitude of diverse conditions, including chronic pain as well as the comorbidities of insomnia, anxiety, and depression [71]. Another systematic review of the literature for studies of CBD used in the treatment of chronic pain retrieved 15 studies, most of which reported that chronic pain was reduced with CBD; in fact, 42% reported less pain intensity with CBD alone and 66% with CBD combined with THC [89].

The studies of CBD for relief of chronic pain have limitations. Many of these studies were small and although they addressed chronic pain, they did not necessarily study tolerability of these agents during prolonged exposure. Some enrolled heterogeneous populations with a diversity of painful conditions. The pain mechanisms and pain etiologies sometimes differed within a systematic review and differed across studies.

Usage patterns of CBD products

Since CBD products are available in many parts of the world without a prescription, people may self-medicate by using them without clinical supervision. For example, people with fibromyalgia often use CBD products either to improve analgesia or to reduce their use of strong prescription analgesics. In an international survey of 878 fibromyalgia patients who reported using cannabinoid products, including, but not limited to, CBD, 72.0% had at times replaced their analgesics with CBD products to manage fibromyalgia symptoms [90]. Of these patients, most reduced or eliminated their use of analgesics or adjuvants, such as NSAIDs (59.0%), opioids (53.3%), gabapentinoids (35.0%), and benzodiazepines (23.1%), replacing them with CBD products. Of those who found CBD to be an effective replacement for prescription pain relievers, more than half permanently switched to CBD, giving up all prescription products with the exception of NSAIDs. When asked to provide reasons for replacing a prescription analgesic with CBD products, the two most commonly stated responses were fewer side effects and better symptom control [90]. Note that the respondents in this survey were under no medical advice to abandon the use of opioids or other products in favor of CBD, but made the decision by themselves to reduce opioid consumption. The use of CBD is common among fibromyalgia patients [91].

A survey audit of patients in New Zealand seeking CBD prescriptions followed 253 patients after they received their CBD prescription for symptoms related to one of four types: chronic noncancer pain (45.6%), neurological pain (15.1%), mental health-related symptoms (16.1%), or cancer symptoms (23.2%). These patients reported a significant improvement in their overall quality of health over baseline and those with nonmalignant pain reported significant analgesic benefit. Significant improvements also occurred in depression and anxiety symptoms, with the positive side effect of better sleep and increased appetite [92]. This survey suggests that CBD offers holistic benefits to pain patients.

A clinical practice guideline on medical cannabis or cannabinoids for chronic pain (noncancer, cancer, neuropathic, nociceptive, nociplastic, mixed, and spasticity) that examined four systematic reviews and included patient values and preferences reported a weak recommendation to trial these products in such patients, based on small improvements in pain, function, sleep quality, as well as their side effects [93]. Further, these authors suggested the use of oral preparations only due to toxins associated with pulmonary exposure. Specifically, they recommended starting with CBD at 5 mg twice daily and increasing to a maximum dose of 40 mg/day, and then adding oral THC if patients reported an insufficient response to CBD [93].

Cannabinoids in context

Neuropathic, Neurogenic, and Neuroinflammatory Pain

CBD inhibits the release of certain neurotransmitters from the pre-synaptic nerve endings which, in turn, modulates post-synaptic neuronal excitation, activating the descending inhibitory pain pathways. This results in a reduction of neural inflammation and oxidative stress [94]. Inflamed tissue actively recruits neutrophils, which can adhere to the endothelium, even to the point that they can rupture the endothelial barrier. Evidence suggests that CBD can reduce pro-inflammatory cytokines, including tumor necrosis factor alpha, IL-1β, and IL-8 [95-97]. CBD can impede the activation cascade of neutrophils and their subsequent degranulation [98]. NADPH oxidase (Nox2) subunits translocate during the oxidative burst and can contribute to neuroinflammation, but CBD appears to disrupt these translocation efforts [98]. In addition, CBD can lower the expression of E-selectin/CD62 from the liver, which plays a key role in endothelial adhesion [99,100]. 

Likewise, CBD inhibits the expression of cyclooxygenase (COX)-1 and COX-2 mRNA expression. COX-1 and COX-2 are involved in the conversion of arachidonic acid into prostaglandins, which sensitize nociceptors to painful stimuli and are generally associated with increased pain intensity. Thus, one of the ways in which CBD reduces pain is by utilizing a mechanism of action similar to that of NSAIDs [101]. Support for this comes from the study by Chrepa et al. who demonstrated the effectiveness of oral CBD to manage acute dental pain, which can also be effectively managed by NSAIDs, in particular, ibuprofen [83,102,103].

Neuropathic, neurogenic pain, and neuroinflammatory syndromes can adversely impact the quality of life and be extremely challenging for clinicians to treat. Twenty-nine people with painful peripheral neuropathy were randomized and treated with a topical CBD product or placebo and then assessed on the Neuropathic Pain Scale. The CBD group showed statistically significant reductions in intense pain, sharp pain, cold sensations, and pruritus with respect to the placebo group. No adverse events were reported [104]. A case series of topical CBD cream used to treat lumbar compression fracture and lower back pain with a neuropathic component found CBD provided significant pain relief [105].

In a systematic review of five randomized controlled trials evaluating the use of cannabinoids for neuropathic pain control, cannabinoids provided significantly greater pain control on the visual analog scale compared to placebo [106]. Another systematic review reported that while CBD products conferred benefits on neuropathic pain patients, there were as yet unquantified potential harms [87]. In a living systematic review and meta-analysis, CBD was found to confer modest analgesic benefits, mainly in the relief of neuropathic pain [107]. In a systematic review of various cannabinoids in the treatment of chronic neuropathic pain, the combination of THC and CBD as well as THC alone offered significant reductions in pain intensity, up to 30%, but the evidence was of moderate-to-low quality [108].

Headaches

Reports about the use of CBD to treat migraine or cluster headaches are limited to mainly case studies or anecdotal reports. However, the analgesic benefits of CBD may well extend to headache disorders [21]. To date, there are no CBD products approved to market for a headache indication [109], but a murine study found that systemically administered CBD could reach brain regions impacted by migraine and likely modulate nociceptive transmission of migraine pain signals [110].

Cannabinoids for Cancer Pain

Cancer pain poses a new paradigm for chronic pain management as the global incidence of cancer is increasing but advances in oncology have made it possible for cancer patients to live for years with “managed disease” or to overcome cancer but have ongoing painful symptoms related to cancer treatments. Chronic cancer-related pain was only recently recognized by the International Classification of Diseases-11 as a medical condition [111]. As much as one-third of patients with cancer-related pain are under-treated or not treated at all for their painful symptoms [112].

The role of cannabinoids in cancer pain has not been thoroughly studied. In a double-blind, placebo-controlled study of 397 patients with different types of advanced cancer, nabiximol was not a statistically significant pain reliever compared to placebo although it did confer benefits with respect to certain quality-of-life metrics at certain study time points. The safety and tolerability of nabiximol were good. Note that a limitation of this nabiximol study was that it enrolled participants with different types of cancer, which may have different pain pathologies [113]. In a double-blind, placebo-controlled study of advanced cancer patients, nabiximol was not statistically superior to placebo in controlling chronic pain otherwise not relieved by optimized opioid treatment [114].

In a systematic review and meta-analysis of 14 studies involving 1,823 participants with cancer pain, it was found that oromucosal nabiximol was not superior to morphine in relieving cancer pain and there was only limited evidence that it was effective in palliative care for cancer pain as a monotherapy [115].

Safety

In assessing the safety of any analgesic, clinicians must weigh benefits against potential side effects, tolerability, adverse events, possible drug-drug interactions, and individual patient preferences. CBD has been less thoroughly studied than THC and, overall, botanical cannabis has a murky and controversial status [116]. Despite this, synthetic CBD is considered to be safe [117]. Due to the limited number and mild nature of most adverse events, CBD is considered overall to be a safe drug, and when more severe adverse events are reported, they tend to be dose dependent [42].

The most notable drug-drug interactions would appear to relate to other drugs that affect the CYP3A4 and CYP2C19 substrates. These drugs would include certain immunosuppressant drugs, chemotherapeutic agents, antiepileptic drugs, antidepressants, and antipsychotics [118]. CBD side effects may include fatigue, drowsiness, sedation, and lethargy [119].

Special Populations

While more research is needed to guide prescribing decisions with respect to CBD and chronic pain, there is a much greater lack of investigation exploring the use of CBD products in special populations, such as pregnant and lactating women, pediatric patients, renally compromised patients, patients with liver disease, and older patients [120]. It is known that cannabinoids may be passed to a nursing child in breast milk, although the effect of cannabinoids on babies and even children has not been well studied [121]. Based on preclinical studies, CBD and THC appear to inhibit embryo implantation and the development of the placenta due to their effects on endometrial receptivity. As such, CBD must be considered potentially risky in the early phases of gestation [122]. In studies of mice, CBD may exert effects on hormonal reproductive functions in both sexes [123]. These findings have not been reported in human studies.

While chronic pain is prevalent in older populations, metabolic issues, potential polypharmacy, the greater incidence of comorbidities, and other conditions in the geriatric population can make analgesic use complex [13]. There are few studies in this specific population that help inform analgesic prescribing choices. Given the limitations of other analgesic options in this older population, the role of CBD may prove to be particularly important.

It is suspected that cannabis use in general may present certain populations with specific risks, such as those with active or familial histories of substance use disorders, those with active psychosis, and those with depressive mood disorders [121]. However, these risk factors have not been thoroughly studied, nor is it clear whether multiple risk factors might exert a synergistic risk to the patient. Such risks are more likely associated with the psychoactive component of the plant in THC rather than CBD.

Multimodal Therapy

Multimodal pain treatment is increasingly used in clinical practice in recognition of the fact that mixed pain syndromes may involve two or more different pain mechanisms which are more effectively treated with analgesics with different mechanisms of action [124]. This, in turn, implies that combination therapy, addressing the neuropathic and nociceptive pain components separately, may provide more comprehensive and life-enhancing relief. However, the effectiveness of multimodal treatments depends on the combination of agents and the types of pain mechanisms involved. In a clinical study of 86 patients with chronic pain due to knee osteoarthritis treated with acetaminophen, the addition of oral CBD 600 mg per day provided no additional analgesic benefit compared to placebo [125]. Of course, it must be noted that acetaminophen was shown in a systematic review and meta-analysis to provide only minimal analgesic benefit for the chronic pain of knee or hip osteoarthritis [126].

Palmitoylethanolamide (PEA) is a fatty acid amide that is known to enhance the actions of endocannabinoids and may be combined with CBD or other related products for chronic pain control [88]. In a systematic review of 16 studies of gynecologic pain patients who used cannabinoids for pain treatments, those who used CBD plus PEA in a multimodal regimen experienced an average visual analog scale pain intensity decrease of 3.35 ± 1.39 [88].

Since CBD may improve cell proliferation and cell migration, it has been suggested that CBD may play an important role in regenerative medicine techniques using mesenchymal stem cells [127]. This is an area that warrants further study.

Perceptions, attitudes, and beliefs about CBD

Patient perceptions about CBD are generally positive. An online survey of 253 outpatients of several California-based pain clinics found 62% had tried CBD to manage their pain, with 59% and 68% reporting that it helped reduce pain and reduce their analgesic consumption, respectively. Three-quarters stated they did not think CBD was harmful and 65% said CBD was not addictive. Slightly more than half of respondents (52%) said they would be comfortable with a prescription CBD product [70]. A survey of 878 fibromyalgia patients found that 72% had substituted CBD products for their other medications and found CBD provided better symptomatic relief with fewer side effects [90]. However, patients using CBD to self-medicate were often unaware of appropriate dosing. About a third of those using CBD products to treat fibromyalgia were unaware of the doses they were taking or what dose they ought to take [91]. This underscores the need for pharmaceutical-grade CBD and greater patient health literacy with respect to CBD and its role in pain therapy.

In a series of interviews with chronic pain patients, the use of CBD and other cannabinoid-related products was found to be related to social acceptability, local availability of the products, affordability, and the guidance of healthcare providers to help inform CBD product choices [128]. A mixed-methods systematic review found that patients who chose CBD products frequently did so in order to reduce the use of analgesics they perceived as more problematic, such as opioids; other factors that influenced CBD use included cost, access, and legal status of the products. Chronic pain patients who use cannabinoids generally prefer oral over inhaled products [129]. In a cross-sectional descriptive survey of 218 veterans receiving care at a single pain clinic, about 10% reported that they used CBD, 52% of whom stated that CBD provided effective analgesia for a variety of painful conditions [130]. In a convenience sample online survey of 428 people with painful symptoms associated with arthritis, 83% stated that CBD use provided pain relief, 66% said CBD improved their physical function, and 66% reported that CBD promoted better sleep. Of the respondents who used CBD, 61% said that CBD allowed them to use fewer other analgesics [131].

Self-medication with over-the-counter CBD products is not always disclosed by patients or considered by physicians in discussing pain with patients [132]. Physicians often will not initiate a discussion or ask about CBD use. Among those specializing in sports medicine, a survey (n = 101) found that 94% believed CBD had a role in managing chronic painful conditions, but only 48% had ever recommended a CBD product to their patients. Respondents also said they did not perceive themselves as knowledgeable about the mechanism of action of CBD (89%) or legal restrictions (66%) [133].

Ongoing clinical research

In a search of ClinicalTrials.com, 63 clinical trials were described involving CBD. Thirty-eight sought to evaluate CBD for chronic painful symptoms and eight studied patients taking CBD to manage cancer pain. The most frequently evaluated patient populations were those with knee osteoarthritis, diabetic neuropathy and other forms of neuropathy, back pain, and unspecified forms of chronic pain. There was a range of conditions in these studies, including endometriosis pain, spinal cord injury, radicular pain, pain in multiple sclerosis, Fabry disease, and bone pain. None of these studies has yet published results and some are still recruiting patients. This suggests the serious scientific interest in CBD and the range of potential applications for these products.

Nabiximol is produced from the botanical form of cannabis in the form of two chemovars, with each clone producing a high level of either CBD or THC. The small molecule is available as an oromucosal spray and while it is cleared to market in Europe, Canada, and other areas, it is not approved for use in the United States. Nabiximol is indicated to treat spasticity and chronic pain, and 15 years of evidence and real-world clinical experience support that it has an acceptable safety profile for this broad indication [134,135].

In an open-label retrospective, flexible-dose study of 655 adults suffering neuropathic back pain and 655 propensity-matched subjects, nabiximol as an add-on treatment was compared to conventional regimens of long-acting oral opioid analgesics. At six months, nabiximol add-on therapy offered tolerability and effectiveness superior to that conferred by the add-on treatment of long-acting oral opioids [136]. Note that this study was not randomized and had no control group.

Discussion

Chronic pain has been rightly described as the “silent epidemic,” in that it describes a population for whom suffering, disability, diminished quality of life, and the risk of mental health disorders are prominent yet who have limited treatment options. The role of cannabinoid analgesics is both a promise and a mirage for those with chronic pain because limited evidence-based arguments can be made for its role in treating chronic pain and many of these products have complicated regulatory and legal status [116]. Despite these limitations, CBD is of great interest to chronic pain patients, many of whom purchase hemp-derived CBD products over-the-counter to help manage chronic painful conditions [116]. Such products are unregulated, and normally consumed through an oral route. Oral administration presents substantial challenges, including difficulties in dose optimization and limited bioavailability. Studies have shown that orally administered CBD undergoes extensive first-pass metabolism in the liver, resulting in high plasma levels of 7-OH-CBD and 7-COOH-CBD metabolites. Additionally, oral CBD administration is associated with high inter- and intrasubject variability in PK parameters. These limitations highlight the urgent need for regulatory-approved cannabinoid products capable of overcoming bioavailability challenges while delivering a long-acting pain relief effect to help manage the various forms of chronic pain, including chronic neuropathic pain, chronic pain with a neuropathic component, neuroinflammatory pain, and cancer pain. New products are needed. Such a product should be a long-acting synthetic cannabinoid, suitable for easy dosing and long-term use in patients who must contend with persistent and often life-altering painful symptoms. A liposomal-CBD injection has been studied in a canine model of osteoarthritis [137,138].

A challenge in this research is that there are multiple products and many of the studies and reports enroll patients with a variety of different pain conditions. The advent of synthetic, long-acting, pharmaceutical-grade CBD for chronic pain will greatly improve our ability to study it coherently because it will be a carefully manufactured product with specific doses and attributes. Future studies of synthetic CBD should enroll patients with chronic pain conditions with the same or highly similar pain pathways. The evidence found to date of the potential role of CBD in treating chronic pain is promising, but more specific evidence is needed to shape prescribing choices and to verify efficacy.

## Conclusions

Synthetic CBD like other forms of cannabinoids is of growing medical interest because of its putative role in chronic pain management. Studies have explored the role of CBD in a variety of indications, including chronic pain with a neuropathic component which can be very challenging to treat. The tolerability and safety of synthetic CBD make it an important product, and it may have a role in multimodal pain regimens. Numerous studies are planned or ongoing to evaluate CBD with promising results.
